# Screening for diabetes with HbA1c: Test performance of HbA1c compared to fasting plasma glucose among Chinese, Malay and Indian community residents in Singapore

**DOI:** 10.1038/s41598-018-29998-z

**Published:** 2018-08-20

**Authors:** Wei-Yen Lim, Stefan Ma, Derrick Heng, E. Shyong Tai, Chin Meng Khoo, Tze Ping Loh

**Affiliations:** 1 Department of Clinical Epidemiology, Tan Tock Seng Hospital, Singapore, Singapore; 2 Epidemiology & Disease Control Division, Ministry of Health, Singapore, Singapore; 3 Public Health Group, Ministry of Health, Singapore, Singapore; 4 Department of Medicine, National University Hospital, Singapore, Singapore; 5 Department Laboratory Medicine, National University Hospital, Singapore, Singapore; 6 Biomedical Institute for Global Health Research and Technology, National University of Singapore, Singapore, Singapore

## Abstract

The prevalence of diabetes in Singapore is high. Screening to facilitate early detection and intervention has been shown to be cost-effective. Current clinical practice guidelines in Singapore recommend screening with fasting plasma glucose (FPG), followed by an oral glucose tolerance test (OGTT) in those with FPG 6.1-6.9 mmol/L. Glycated haemoglobin A1c (HbA1c) has robust stability at ambient temperature, and can be performed on non-fasted capillary blood samples, making it an attractive potential alternative for screening. However, limitations of HbA1c include differential performance in different races, and its performance as a screening test has not been well characterized in Asian populations. This study compares HbA1c and FPG as diabetes screening modalities in 3540 community-dwelling Singapore residents of Chinese, Malay and Indian race to detect diabetes mellitus diagnosed based on blood glucose (FPG ≥ 7.0 mmol/L, 2 hr OGTT ≥ 11.1 mmol/L). The area under the receiver-operating-characteristic curve (AUC) was higher for FPG compared to HbA1c in the overall population and age, race and age-race strata, but these differences were not statistically significant. HbA1c > = 7.0% identified 95% of individuals with diabetes mellitus, and the remainder had impaired glucose tolerance (IGT). HbA1c cut-off at 6.1% had better sensitivity (0.825) to FPG at 6.1 mmol/L. The positive predictive value of HbA1c at 6.1% was 40–50% in different age-race combinations with a negative predictive value of about 98%. If follow-up screening with FPG is used, a lower cut-off at 5.6 mmol/L is appropriate in identifying people with pre-diabetes, as about 85% of people with HbA1c 6.1–6.9% and FPG 5.6–6.9 mmol/L had IFG/IGT or diabetes in the study sample. HbA1c is an appropriate alternative to FPG as a first-step screening test, and the combination of Hba1c > = 6.1% and FPG > = 5.6 mmol/L would improve the identification of individuals with diabetes mellitus and prediabetes.

## Introduction

Several organizations around the world have recommended that diabetes mellitus can be diagnosed using one of the following tests: glycated haemoglobin A1c (HbA1c), fasting plasma glucose (FPG) or oral glucose tolerance test (OGTT). The advantages of using HbA1c for diagnosis of diabetes include its robust stability at ambient temperature, and the ability to use non-fasting, random blood samples, allowing this test to be performed at any time of the day^1^. However, the use of HbA1C for the diagnosis of diabetes has not been adopted universally. In the Asia-Pacific region, Malaysia accepts the use of HbA1c, but at a lower cut-off of 6.3% or higher^2^, while New Zealand uses a higher cut-off at 50 mmol/mol, or 6.7%^3^. In Singapore, we have recommended against the use of HbA1c for the diagnosis of diabetes^4^ due to concerns around the potential impact of race/ ethnicity^5^, the presence of haemoglobin variants and other conditions that affect red cell turnover (including the recent observation that G6PD deficiency, a condition that is common in Singapore, has an effect on HbA1c independent of glycaemia)^6^. In fact, in the 2018 standards of medical care in diabetes, the American Diabetes Association (ADA) has added additional recommendations to clarify the appropriate use of the HbA1c test generally and in the diagnosis of diabetes in the abovementioned clinical situations^7^. Thailand likewise has not used HbA1c to diagnose diabetes^8^.

Despite the limitations described in the preceding paragraphs, HbA1c is highly correlated with blood glucose^7^. While Singapore does not use HbA1c to diagnose diabetes, this does not preclude using it as a screening test for diabetes in asymptomatic individuals, as is recommended by other health authorities^9,10^. However, the appropriate HbA1c cut-off when this is used as a screening test for diabetes is not clear. While the World Health Organization (WHO) and American Diabetes Association (ADA) recommends HbA1c 6.5% as an appropriate cut-off to diagnose diabetes mellitus^7,11^, they have also noted that at this cut-off, HbA1c is not very sensitive, and HbA1c values lower than 6.5% does not exclude diabetes, suggesting that this cut-off is not suitable for use in screening. A systematic review conducted in 2007 summarising findings from cross-sectional studies comparing HbA1c and FPG with OGTT as gold-standard noted that most studies reported that HbA1c was comparable to FPG in performance and proposed 6.1% as a receiver operating characteristic curve (ROC)-optimal cut-off. The study noted however that population-specific cut-offs might be necessary given ethnic differences in sensitivity and specificity of HbA1c likely linked to genetic variation in haemoglobin concentration, lifespan of red blood cells, and rates of glycation^12^. Further, the different prevalence of diabetes in different populations, and other population-specific factors may also influence the choice of cut-off.

Furthermore, the limitations of HbA1c as described earlier could be addressed if it is followed by a blood glucose test for confirmation. At this time, the ADA recommends that, in an asymptomatic individual, if HbA1c is > = 6.5%, the same test can be repeated to confirm the diagnosis. However, if the second test is a measure of blood glucose (fasting plasma glucose or oral glucose tolerance test) rather than HbA1c, it may help address some of the limitations of HbA1c. This study thus also provides an opportunity to determine an appropriate threshold for fasting plasma glucose as a follow-up test for identifying those with diabetes or pre-diabetes. The ADA has recommended lowering the cut-off for impaired fasting glucose from 6.1 to 5.6 mmol/L. We found that locally, among the group with FPG 5.6–6.0 mmol/L, it was only those who also had impaired glucose tolerance (IGT) who were at greatest risk of diabetes and cardiovascular disease^13^. However, our current screening strategy (only those with FPG 6.1–6.9 mmol/L would undergo an OGTT as follow-up test) would miss a significant number of individuals in this group, in whom diabetes prevention has been demonstrated to be effective in randomized controlled trials. Using FPG in combination with HbA1c may allow us to identify a larger proportion of individuals with IGT.

The aims of this study are to evaluate the use of HBA1c for diabetes screening, to determine the optimal HbA1c cut-off for screening for diabetes, and to assess if HbA1c could be combined with FPG to detect individuals with diabetes mellitus and impaired glucose tolerance, in a multiracial population living in Asia where the prevalence of diabetes mellitus is high.

## Methods

### Study subjects

This study used data from the cross-sectional National Health Survey collected between 17 March 2010 and 13 June 2010. All participants provided written informed consent for further analysis of the collected data. This study design received ethics board approval (Medical & Dental Board, Health Promotion Board, ref: 005/2009). Details of this survey are available in the official report^14^. The methods described in this study were performed according to relevant local guidelines and regulation.

Briefly, the National Health Survey employed a two-phase sampling strategy. In the first phase, a 2-stage sampling design was used, where geographical zones within 5 km of the six study sites were first selected, and then 47,500 residential dwelling units were randomly selected without replacement. A random sample of 17,000 dwellings was then selected for house visits to enumerate all household members. In the 2nd phase, a random sample of 7,695 individuals was selected from the enumeration list, using a disproportionate stratified sampling design, with oversampling of racial minorities (30% Chinese, 30% Malays, 30% Indians and 10% other races). Race was defined using criteria set by the Ministry of Home Affairs, Singapore: classification of race occurs at birth or the point of naturalization, and people take the race of their birth father. In the sample, 183 persons were ineligible for reasons such as pregnancy and recent delivery, institutionalisation, death, and being overseas during the entire duration of the study period. Of the 7,512 eligible individuals aged 18 to 79 years, 4,337 participated in the survey (participation rate of 57.7%).

### Laboratory analysis

Blood samples from participants were collected after an overnight fast of at least ten hours, using standard phlebotomy procedure. The OGTT was performed by administering 75 g of anhydrous glucose (Trutol), and plasma glucose concentration was measure again two hours later. Plasma glucose and HbA1c were measured on the Roche Modular platforms. The HbA1c immunoassay used was National Glycohemoglobin Standardization Program-certified.

### Definition of glycaemic status

The glycaemic status of the participants was defined according to those recommended by World Health Organization in 2006^15^. Normal FPG was defined as <6.1 mmol/L while impaired FPG was defined as 6.1 mmol/L to 6.9 mmol/L. Normal OGTT was defined as ≤7.7 mmol/L and impaired OGTT was defined as 7.8 mmol/L to 11.0 mmol/L. Diabetes was defined as having any of the following: FPG ≥ 7.0 mmol/L or OGTT ≥ 11.1 mmol/L.

### Exclusion criteria

Only subjects who did not complete the FPG (n = 174), OGTT (n = 794) and HbA1c measurements (n = 54) were excluded from analysis. Participants who had diabetes and were on diabetic medication did not undertake the OGTT. In total, the final sample for this study was 3,540.

### Statistical analysis

Demographic and laboratory variables were tabulated and summarised, weighted by sample weights including unequal probability of selection, non-response and post-stratification. Using the WHO 2006 definition for diabetes (FPG ≥ 7.0 mmol/l or OGTT ≥ 11.1 mmol/L), the Receiver Operating Characteristic (ROC) curve was calculated for FPG and HbA1c as a first screening test for diabetes mellitus. The tests of equality of Area Under the Curve of the two ROCs (AUCs) were effected with the roccomp command in Stata, using a χ2 test. The sensitivity and specificity of FPG at ≥5.6 mmol/L, ≥6.1 mmol/L, and ≥ 7.0 mmol/L, and HbA1c at cut-offs between 5.7% and 6.5% and at 7.0% were calculated, weighted by sample weights. To further examine the diagnostic performance of the laboratory tests in the community setting, the Positive Predictive Value and Negative Predictive Value at diagnostic thresholds for HbA1c and FPG were calculated using the 2016 mid-year population estimates provided by the Departments of Statistics Singapore^16^, and age- and race-matched prevalence of diabetes^14^. Analyses were repeated with stratification by age (<40 years, and 40 years and older), gender and race (Chinese, Malays, Indians). All analyses were performed on Stata 14.0.

### Data Availability

The data contained in this submission belongs to the Ministry of Health and is not available for public access under local regulations. Interested party can contact Dr. Stefan Ma (Stefan_MA@moh.gov.sg).

## Results

In all, 3,540 subjects were included in the final analysis. The demographic and laboratory parameters of these subjects are summarised in Table 1. Overall, 7.6% of the study population had FPG and/or 2hr-OGTT in the diabetic range as defined by WHO 2006 criteria.

**Table 1:** Summary of demographic and laboratory parameters of subjects included in the final analysis.

Overall		3540	
			**Weighted mean (SD)**
HbA1c			5.79 (0.64)
Fasting Plasma Glucose			5.31 (1.06)
2 hr Oral Glucose Tolerance Test			6.85 (2.96)
		**Number of subjects**	**Weighted prevalence (%)**
Age	Mean (SD)	42.5 (14.5)	
	<40 years	1563	44.5
	40–64 years	1722	47.8
	≥65 years	255	7.8
Gender	Male	1671	47.6
	Female	1869	52.4
Ethnic group	Chinese	1164	76.8
	Malay	1011	11.5
	Indians	1082	8.5
	Others	283	3.2
FPG	<7.0 mmol/L	3357	96.3
	≥7.0 mmol/L	183	3.7
OGTT	<11.1 mmol/L	3237	93.1
	≥11.1 mmol/L	303	6.9
Diabetes	Fasting glucose <7.0 mmol/L and OGTT <11.1 mmol/L	3208	92.4
	Fasting glucose ≥7.0 mmol/L or OGTT ≥11.1 mmol/L	332	7.6

The mean values for FPG and HbA1c were slightly lower in Chinese (FPG 5.26 mmol/L, HbA1c 5.77%) compared to Malays (FPG 5.54 mmol/L, HbA1c 5.90%) and Indians (FPG 5.46 mmol/L, HbA1c 5.89%), while the dispersion of both FPG and HbA1c appeared smaller and thus more tightly clustered around the mean for Chinese compared to other two races (standard error for FPG 0.027 and for HbA1c 0.017, compared to 0.057 and 0.032 respectively for Malays, and 0.046 and 0.027 for Indians), see Supplementary Figs 1 and 2.

**Figure 1:**
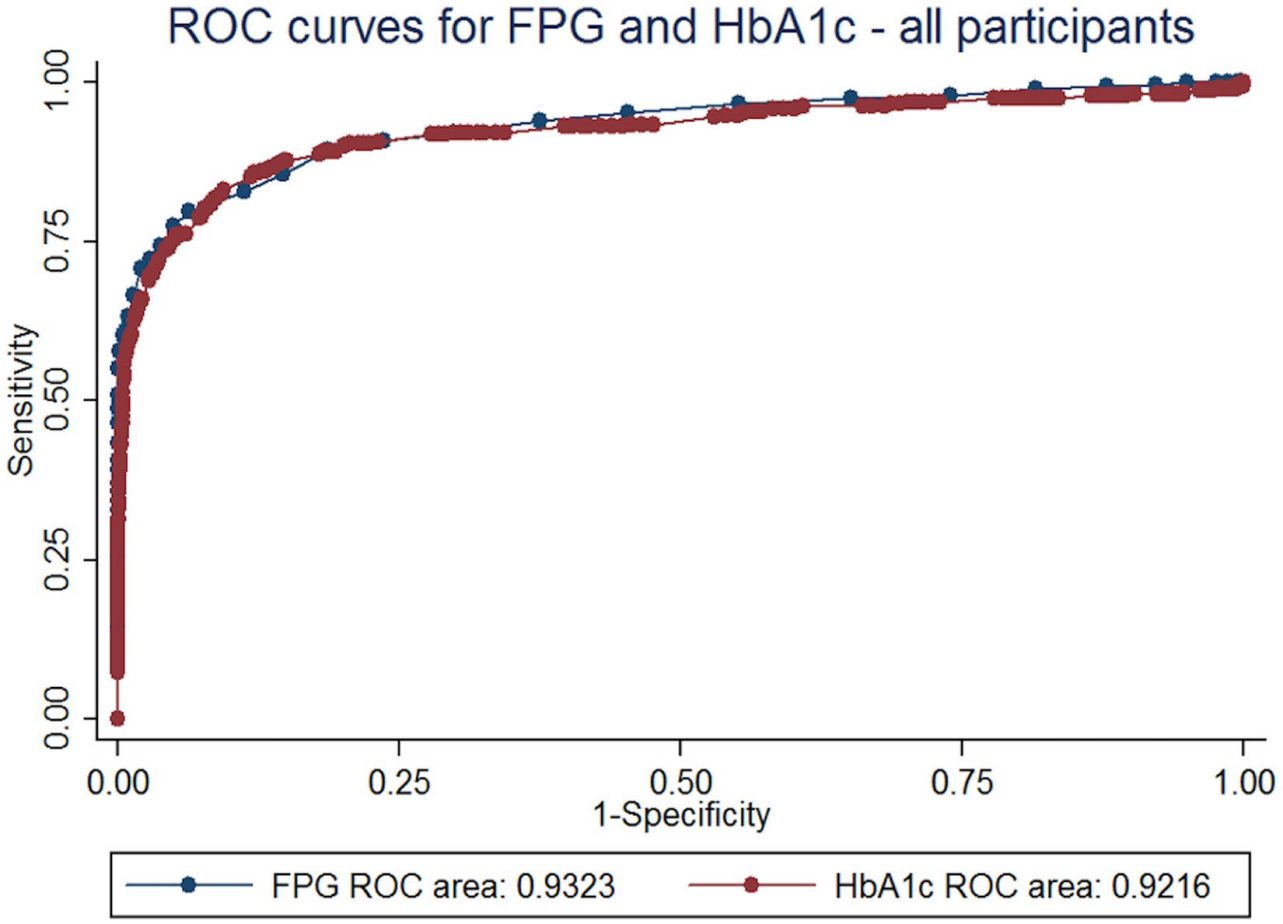
Receiver operating characteristic (ROC) curves for HbA1c and FPG in all study participants.

**Figure 2:**
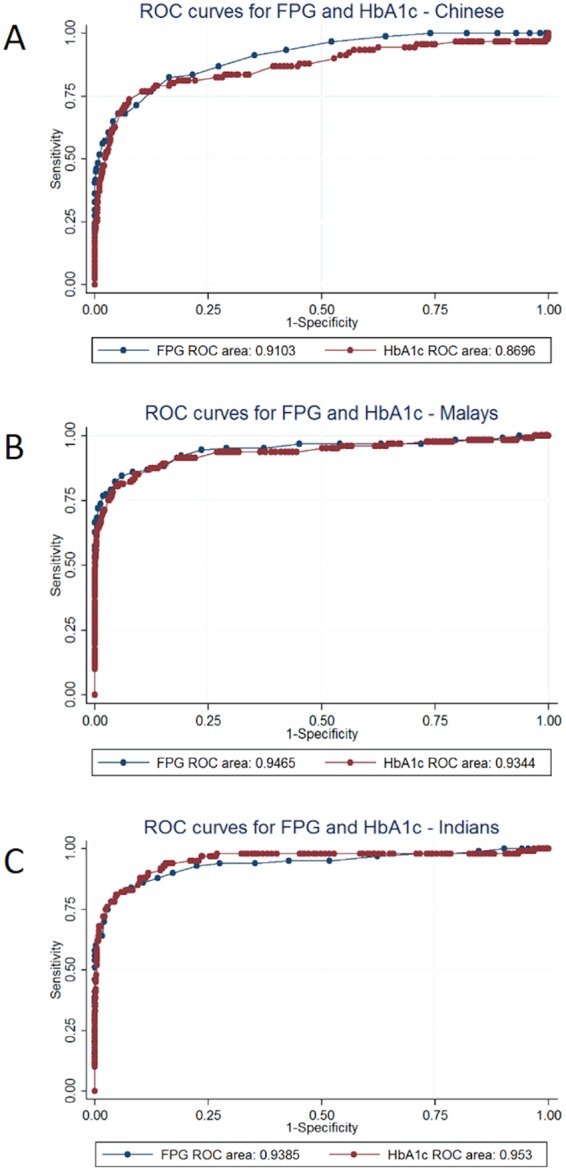
Receiver operating characteristic (ROC) curves for HbA1c and fasting plasma glucose (FPG) in (**A**) Chinese (**B**) Malay (**C**) Indian participants.

The ROC curves for FPG and HbA1c overall and among Chinese, Malays and Indians against the WHO glucose-only criteria for diabetes are shown in Figs 1 and 2. The AUCs of FPG and HbA1c and p value for equality in AUC for different ages, genders, races and age-race combinations are presented in Table 2. Overall, the AUC was higher for FPG compared to HbA1c, both overall and in analyses stratified by age, gender, race and age-race combinations. The AUCs for FPG and HbA1c were lowest for the Chinese compared to other races. Indians, unlike Chinese and Malays, had higher AUC for HbA1c. The lowest AUC observed for HbA1c in the different groups tested was 0.842 in Chinese 40 years and older. None of the differences in AUC between HbA1c and FPG were statistically significant, suggesting that FPG and HbA1c performed similarly in classifying patients using the WHO 2006 criteria for diabetes mellitus, both overall, and for various age-race combinations.

**Table 2:** Sensitivity and specificity of various cut-offs of fasting glucose and HbA1c compared to glucose-only WHO criteria for diabetes*, overall, and by age, gender, ethnicity and age-ethnicity combinations.

	Overall	< 40 years	> = 40 years	Males	Females	Chinese	Ma®lays	Indians	Chinese < 40 years	Chinese > = 40 years	Malay < 40 years	Malay > = 40 years	Indian < 40 years	Indian > = 40 years
Area under the ROC (AUC) for fasting plasma glucose	0.932	0.963	0.910	0.932	0.932	0.910	0.947	0.939	0.992	0.875	0.961	0.931	0.945	0.924
AUC for HbA1c	0.922	0.930	0.899	0.928	0.914	0.870	0.934	0.953	0.849	0.842	0.889	0.925	0.989	0.928
P value for equality of AUCs	0.34	0.32	0.40	0.80	0.31	0.12	0.44	0.45	0.32	0.25	0.23	0.72	0.12	0.89

*Fasting Plasma Glucose ≥ 7.0 mmol/L or 2 hr post glucose load ≥ 11.1 mmol/L.

Table 3 presents sensitivity, specificity, positive and negative predictive values in the 3 major races for FPG at ≥5.6 mmol/l and ≥6.1 mmol/l, and for HbA1c between 5.7% and 6.5% at 0.1% intervals, and at 7.0%. The positive predictive values of both HbA1c and FPG were consistently the highest for the Malays and the lowest for the Chinese, while the negative predictive values were largely similar among the races. For the overall population, the sensitivity was 0.692 and specificity 0.963 at a cut-off of FPG 6.1 mmol/l or more. HbA1c cut-offs at 6.1–6.3% would have comparable performance in terms of sensitivity (0.679–0.825) and specificity (0.903–0.969). In particular, a HbA1c cut-off of ≥ 6.1% would have much greater sensitivity of 0.825 (and better negative predictive value in the 3 races) compared to FPG at ≥ 6.1 mmol/l. At HbA1c 7.0%, the positive predictive value was 0.92–0.977 for the 3 races, with the remainder having impaired glucose tolerance, suggesting that this cut-off could be used to “rule-in” diabetes mellitus without need for further testing.

**Table 3:** Sensitivity, specificity positive predictive value and negative predictive at cut-offs of Fasting Plasma Glucose (FPG) at 5.6 mmol/L and 6.1 mmol/L, and HbA1c between 5.7% and 6.5% for overall, Chinese, Malays and Indians, compared to glucose-only WHO criteria for diabetes*.

		Overall	Chinese	Malays	Indians
Fasting plasma glucose ≥ 5.6 mmol/L	Sensitivity	0.872	0.855	0.929	0.910
	Specificity	0.841	0.847	0.824	0.793
	Positive predictive value		0.305	0.405	0.345
	Negative predictive value		0.985	0.988	0.984
Fasting plasma glucose ≥ 6.1 mmol/L	Sensitivity	0.692	0.638	0.849	0.800
	Specificity	0.963	0.966	0.958	0.949
	Positive predictive value		0.588	0.719	0.636
	Negative predictive value		0.969	0.979	0.972
HbA1c ≥ 5.7%	Sensitivity	0.923	0.903	0.958	0.990
	Specificity	0.502	0.497	0.537	0.465
	Positive predictive value		0.127	0.213	0.185
	Negative predictive value		0.983	0.185	0.996
HbA1c ≥ 5.8%	Sensitivity	0.902	0.879	0.940	0.990
	Specificity	0.633	0.629	0.670	0.597
	Positive predictive value		0.159	0.267	0.228
	Negative predictive value		0.984	0.988	0.997
HbA1c ≥ 5.9%	Sensitivity	0.878	0.845	0.940	0.990
	Specificity	0.759	0.760	0.749	0.722
	Positive predictive value		0.218	0.323	0.292
	Negative predictive value		0.983	0.989	0.998
HbA1c ≥ 6.0%	Sensitivity	0.851	0.819	0.921	0.961
	Specificity	0.853	0.859	0.829	0.821
	Positive predictive value		0.310	0.408	0.375
	Negative predictive value		0.982	0.987	0.993
HbA1c ≥ 6.1%	Sensitivity	0.825	0.798	0.883	0.912
	Specificity	0.903	0.906	0.889	0.874
	Positive predictive value		0.395	0.504	0.445
	Negative predictive value		0.981	0.982	0.986
HbA1c ≥ 6.2%	Sensitivity	0.746	0.709	0.844	0.860
	Specificity	0.945	0.948	0.926	0.925
	Positive predictive value		0.511	0.588	0.559
	Negative predictive value		0.975	0.978	0.980
HbA1c ≥ 6.3%	Sensitivity	0.679	0.621	0.833	0.834
	Specificity	0.969	0.972	0.955	0.957
	Positive predictive value		0.622	0.700	0.677
	Negative predictive value		0.968	0.977	0.978
HbA1c ≥ 6.4%	Sensitivity	0.580	0.498	0.797	0.796
	Specificity	0.979	0.980	0.972	0.975
	Positive predictive value		0.650	0.777	0.764
	Negative predictive value		0.958	0.972	0.973
HbA1c ≥ 6.5%	Sensitivity	0.509	0.425	0.723	0.729
	Specificity	0.988	0.987	0.985	0.988
	Positive predictive value		0.712	0.857	0.849
	Negative predictive value		0.953	0.963	0.964
HbA1c ≥ 7.0%	Sensitivity	0.335	0.258	0.567	0.501
	Specificity	0.998	0.998	0.998	0.998
	Positive predictive value		0.920	0.977	0.955
	Negative predictive value		0.940	0.944	0.937

*FPG ≥ 7.0 mmol/L or 2 hour Oral Glucose Tolerance Test ≥ 11.1 mmol/L.

In Table 4, we considered the use of FPG as a follow-up test for those persons with HbA1c 6.1–6.9%, using the 2 thresholds of FPG 5.6 mmol/l or FPG 6.1 mmol/l, and evaluating their diabetes status using WHO glucose-only criteria (normal status defined as FPG < 6.1 mmol/l AND 2hr-OGTT ≤ 7.7 mmol/l; IFG/IGT status defined as FPG 6.1–6.9 mmol/l OR 2hr-OGTT 7.8–11.0 mmol/l. Diabetes status defined as FPG ≥ 7.0 mmol/l OR 2hr-OGTT ≥ 11.1 mmol/l). Among those with HbA1c 6.1–6.9% but FPG < 5.6 mmol/l, about half (48.8%) are normal, and only 7.7% would be diabetics. Among those with HbA1c 6.1%–6.9% but FPG ≥ 6.1 mmol/l, more than half (58.9%) would have diabetes. Among those with HbA1c 6.1–6.9% but FPG ≥ 5.6 mmol/l, only 13.5% would have normal status, and 43.1% would be diabetics while 43.4% have IGT.

**Table 4:** Diabetes status, as defined using WHO glucose-only criteria, in those with HbA1c 6.1–6.9%, stratified by FPG classes. Normal status defined as FPG < 6.1 mmol/l AND 2hr-OGTT ≤ 7.7 mmol/l; IFG/IGT status defined as FPG 6.1–6.9 mmol/l OR 2hr-OGTT 7.8–11.0 mmol/l; Diabetes status defined as FPG ≥ 7.0 mmol/l OR 2hr-OGTT ≥ 11.1 mmol/l.

HbA1c Screening with follow-up FPG	Diabetes status*	Weighted proportion within each HbA1c/FPG group
HbA1c 6.1–6.9%, FPG < 5.6 mmol/l	Normal	48.8%
	IFG/IGT	43.5%
	Diabetes	7.7%
HbA1c 6.1–6.9%, FPG 5.6–6.0 mmol/l	Normal	25.6%
	IFG/IGT	45.5%
	Diabetes	28.9%
HbA1c 6.1–6.9%, FPG > = 6.1 mmol/l	Normal	0%
	IFG/IGT	41.1%
	Diabetes	58.9%
HbA1c 6.1–6.9%, FPG > = 5.6 mmol/l	Normal	13.5%
	IFG/IGT	43.4%
	Diabetes	43.1%

## Discussion

The use of HbA1c is premised upon a relatively constant glycation rate that reflects the ambient glucose condition. However, this assumption may be violated under several conditions^11^. They include biological factors that can alter the relationship between ambient glucose concentration and HbA1c, and analytical factors that can affect its accurate measurement. The biological factors include clinical conditions that alter erythropoiesis, glycation rate, erythrocyte destruction and altered haemoglobin. More recently, genetic determinants of HbA1c have also been described^6,17^. Analytical interferences such as hyperbilirubinaemia, carbamylated haemoglobin, certain medications and haemoglobin variants can also cause spuriously high or low HbA1c results. Analytical interferences are method-specific and should be kept in mind when interpreting laboratory results. In this study, the HbA1c was measured by an immunoassay method which was not affected by haemoglobin variants that are common in this population^18,19^. Indeed, although thalassaemias (esp β-thalassaemia) are most common among the Malay population in Singapore^20^, we did not see a substantially lower AUC for HbA1c in Malays compared to other races.

Our results suggest that FPG and HbA1c have similar performance as screening modalities for diabetes in a multi-racial Asian population, in relation to their ability to discriminate those who do, and do not, have diabetes mellitus based on blood glucose measurements (whether fasting or OGTT). Taken together with the greater pre-analytical stability, lower day-to-day perturbation caused by acute events like stress or illness, and the fact that the test can be conducted in the non-fasting state, HbA1c appears to be a suitable alternative to FPG for population screening for diabetes mellitus.

Our data suggests that an HbA1c cut-off at 6.1%–6.3% would have comparable sensitivity (0.679–0.825) and specificity (0.903–0.969) to the cut-off for FPG at 6.1 mmol/l that is currently recommended by Singaporean health authorities (sensitivity of 0.692, specificity of 0.963) as the first stage in screening for diabetes^21^. For screening, we suggest a more sensitive cut-off at the expense of more false positives would be preferable, especially since the “cost” of a false positive in this context is relatively low (i.e. use of confirmatory OGTT or FPG test). A cut-off of HbA1c at 6.1% or greater appears to be reasonable, with significant improvement in sensitivity with an acceptable decrease in specificity. The positive predictive value of HbA1c at 6.1% would be 40–50%, with a high negative predictive value of >98%. Compared to FPG at a cut-off of 6.1 mmol/l, HbA1c at 6.1% or greater would identify an additional 13% of people with diabetes if everyone in Singapore were screened (82.4% of all people with diabetes would be identified compared to 69.2%) (see Supplementary Table 1).

Our proposal, that the threshold used for screening should be lower than that recommended for diagnosis of diabetes, is consistent with the findings of others. A Japanese group proposed a cut-off at 6.0% if it were used as the first-step screening test with glucose-based confirmation in a Japanese population^22^, and in their study, this cut-off had a sensitivity of 0.837 and specificity of 0.876. A recent Chinese study proposed an HbA1c cut-off at 6.3% to screen for diabetes; this achieved sensitivity of 0.67 and specificity of 0.81 against glucose-only criteria for diagnosing diabetes^23^. A 2010 study in Australia proposed a threshold of 5.5% to rule-out diabetes in their population^24^.

While we recognize, and have shown, that HbA1c effectively discriminates between those with and without diabetic retinopathy in our population^25^, which formed the basis for the recommendations to adopt HbA1c for the diagnosis of diabetes mellitus in other populations, individuals can have marked discrepancies between HbA1c and blood glucose. In their most recent recommendations, the ADA recommended that marked discrepancies between measured HbA1c and plasma glucose levels should prompt consideration that the HbA1c assay might not be reliable for that individual. However, they stopped short of recommending a second confirmatory test that utilized blood glucose. In contrast, Japan uses HbA1c at a cut-off of 6.5%, but does not permit making a diagnosis of diabetes on the basis of HbA1c alone and mandates follow-up glucose-based testing^26^.

In this regard, beyond the main question we had about the use of HbA1c as an alternative to FPG in screening, we asked two additional questions in this study. Firstly, we asked if there was a level of HbA1c beyond which additional testing with blood glucose was likely to be unnecessary. Our data showed that among those with HbA1c > = 7.0%, 94% had diabetes mellitus based on fasting glucose or OGTT, while the remainder had IGT, suggesting that this level of HbA1c was a reasonable level at which to “rule-in” the disease. This is consistent with the proposal by Lu et at in Australia to use an HbA1c threshold > = 7% to likewise “rule-in” diabetes mellitus^24^. It would be reasonable to treat these individuals as if they had diabetes mellitus without further testing, although we recommend that this needs to be guided by clinical judgment and follow-up tests may be indicated, for example, if capillary glucose measurements or symptoms suggest the occurrence of hypoglycaemia with treatment.

Secondly, we examined the use of fasting plasma glucose as a follow-up test to HbA1c to confirm the presence of diabetes mellitus, and evaluated the impact of adopting a lower threshold for the diagnosis of impaired fasting glucose from 6.1 mmol/l to 5.6 mmol/l (as recommended by the ADA and the IDF). We had previously decided not to adopt this threshold because it would result in the diagnosis of impaired fasting glucose in a large proportion (about 20%) of the population, of which only a small subset (20%) had IGT and represented the group that was at the greatest risk of future diabetes and CVD. We were also cognizant that all trials of diabetes prevention had been conducted in individuals with IGT. The diagnosis of pre-diabetes in a large proportion of the population, amongst whom many have a lower risk of diabetes than those who had been included in randomized controlled trials, will greatly increase the cost, and reduce the cost-effectiveness, of diabetes prevention at the population level.

We found that if an individual had HbA1c 6.1%–6.9%, a fasting plasma glucose ≥5.6 mmol/l identified a group of individuals in whom 43.1% would have diabetes mellitus based on an oral glucose tolerance test, and a further 43.4% had impaired glucose tolerance (see Table 4). As such, in the context of an elevated HbA1c, FG > 5.6 mmol/l represents a group at high risk of current and future diabetes. It would be appropriate to recommend diabetes prevention intervention for this group with HbA1c 6.1–6.9% and FPG 5.6–6.9 mmol/l (FPG ≥ 7.0 mmol/l would be in the diabetic range and should be managed separately) that focused on therapeutic lifestyle modification and/or treatment with metformin. Furthermore, the overall prevalence of HbA1c > = 6.1–6.9% AND FPG 5.6–6.9 mmol/l in the population is only 6.9%, compared to using FPG alone to diagnose IFG (FPG 5.6–6.9 mmol/l), which would label 17.7% of the population as pre-diabetic.

A limitation of this study is that we only performed OGTT once. Screening guidelines largely require repeat tests in otherwise asymptomatic individuals. The true prevalence of diabetes is likely lower than that based on a single test^27^. We believe that this limitation should have minimal impact on the AUC for FPG (since FPG forms part of the glucose-based definition of diabetes that we used), while the AUC for HbA1c might be better than estimated in this study (since HbA1c values are known to be more consistent and stable). Another limitation is that we did not consider the economics of using HbA1c versus FPG as screening modalities, with the necessity of doing repeat OGTTs for screen-positive cases. Economic modelling of the 2 different screening strategies (of using HbA1c versus FPG) at various cut-offs would be needed to establish the likely cost impact that would result from a switch from FPG to HbA1c as the primary screening test. The key strength of our study is that we had OGTT tests on all participants. Further, we oversampled racial minorities and older individuals, thus allowing us to investigate with sufficient precision the use of HbA1c as a screening tool in a multiracial population across the age spectrum.

In conclusion, in a multiracial Asia population with a high prevalence of diabetes mellitus, HbA1c is a suitable alternative to FPG as a test for screening for diabetes mellitus. To avoid diagnosing diabetes in individuals with elevated HbA1c but normal blood glucose, we recommend a follow-up test with glucose measurement. If the follow-up test is FPG, then a cut-off of > = 5.6 mmol/l would identify a group of individuals in whom the vast majority would be suitable for diabetes prevention or treatment intervention with therapeutic lifestyle modification or metformin.

## Electronic supplementary material

Supplementary Information
