# Supplementary material for: Screening for diabetes with HbA1c: Test performance of HbA1c compared to fasting plasma glucose among Chinese, Malay and Indian community residents in Singapore
Source: Sci Rep. 2018 Aug 20;8:12419. doi: 10.1038/s41598-018-29998-z (PMC6102285; doi:10.1038/s41598-018-29998-z)

Supplementary information

Title: Screening for diabetes with HbA1c: Test performance of HbA1c compared to fasting plasma glucose among Chinese, Malay and Indian community residents in Singapore

Wei-Yen Lim,^1^ Stefan Ma,^2^ Derrick Heng,^3^ E Shyong Tai,^4^ Chin Meng Khoo,^4^ Tze Ping Loh^5,6*^

Affiliations:

^1^Department of Clinical Epidemiology, Tan Tock Seng Hospital, Singapore.

^2^Epidemiology & Disease Control Division, Ministry of Health, Singapore.

^3^Public Health Group, Ministry of Health, Singapore

^4^Department of Medicine, National University Hospital, Singapore

^5^Department Laboratory Medicine, National University Hospital, Singapore

^6^Biomedical Institute for Global Health Research and Technology, National University of Singapore, Singapore

Supplementary Table 1: Expected screening status using Fasting Plasma Glucose (FPG) and HbA1c of all persons with diabetes, impaired fasting glucose/impaired glucose tolerance, and normal glucose using WHO glucose-only criteria.

| **Diabetes status*** | **Weighted proportion in population** | **Screening category (FPG and Hba1c)** | **Weighted proportion within each DM status group** |
| --- | --- | --- | --- |
| Normal | 75.07% | FG<6.1, HbA1c<6.1% | 95.5% |
|  |  | FG=>6.1mmol/l, HbA1c<6.1% | 0 |
|  |  | FG<6.1mmol/l, HbA1c=>6.1% | 4.5% |
|  |  | FG=>6.1mmol/l, Hba1c=>6.1% | 0 |
| IFG/IGT | 17.29% | FG<6.1, HbA1c<6.1% | 57.58% |
|  |  | FG=>6.1mmol/l, HbA1c<6.1% | 9.9% |
|  |  | FG<6.1mmol/l, HbA1c=>6.1% | 22.81% |
|  |  | FG=>6.1mmol/l, Hba1c=>6.1% | 9.72% |
| Diabetic | 7.64% | FG<6.1, HbA1c<6.1% | 9.5% |
|  |  | FG=>6.1mmol/l, HbA1c<6.1% | 8.04% |
|  |  | FG<6.1mmol/l, HbA1c=>6.1% | 21.28% |
|  |  | FG=>6.1mmol/l, Hba1c=>6.1% | 61.19% |

*****Normal status defined as FPG <6.1 mmol/l AND 2 hour-OGTT ≤7.7 mmol/l; IFG/IGT status defined as FPG 6.1-6.9 mmol/l OR 2hr-OGTT 7.8-11.0 mmol/l; Diabetes status defined as FPG ≥7.0 mmol/l OR 2hr-OGTT ≥11.1 mmol/l.

Supplementary Figure 1: Distribution of fasting glucose values in the study sample, stratified by race with normal density plot superimposed.


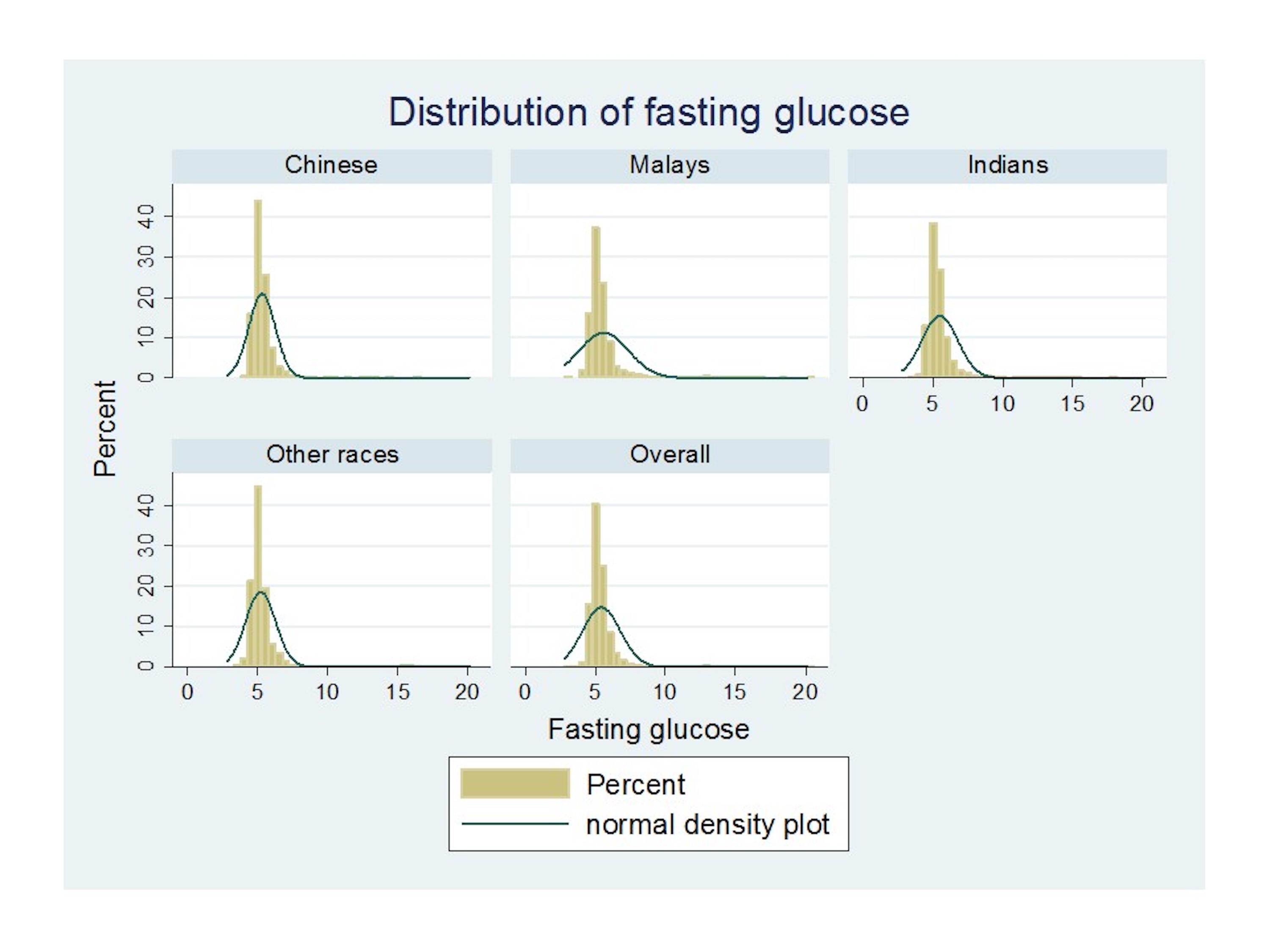


Supplementary Figure 2: Distribution of glycated haemoglobin A1c (HbA1c) values in the study sample, stratified by race with normal density plot superimposed.


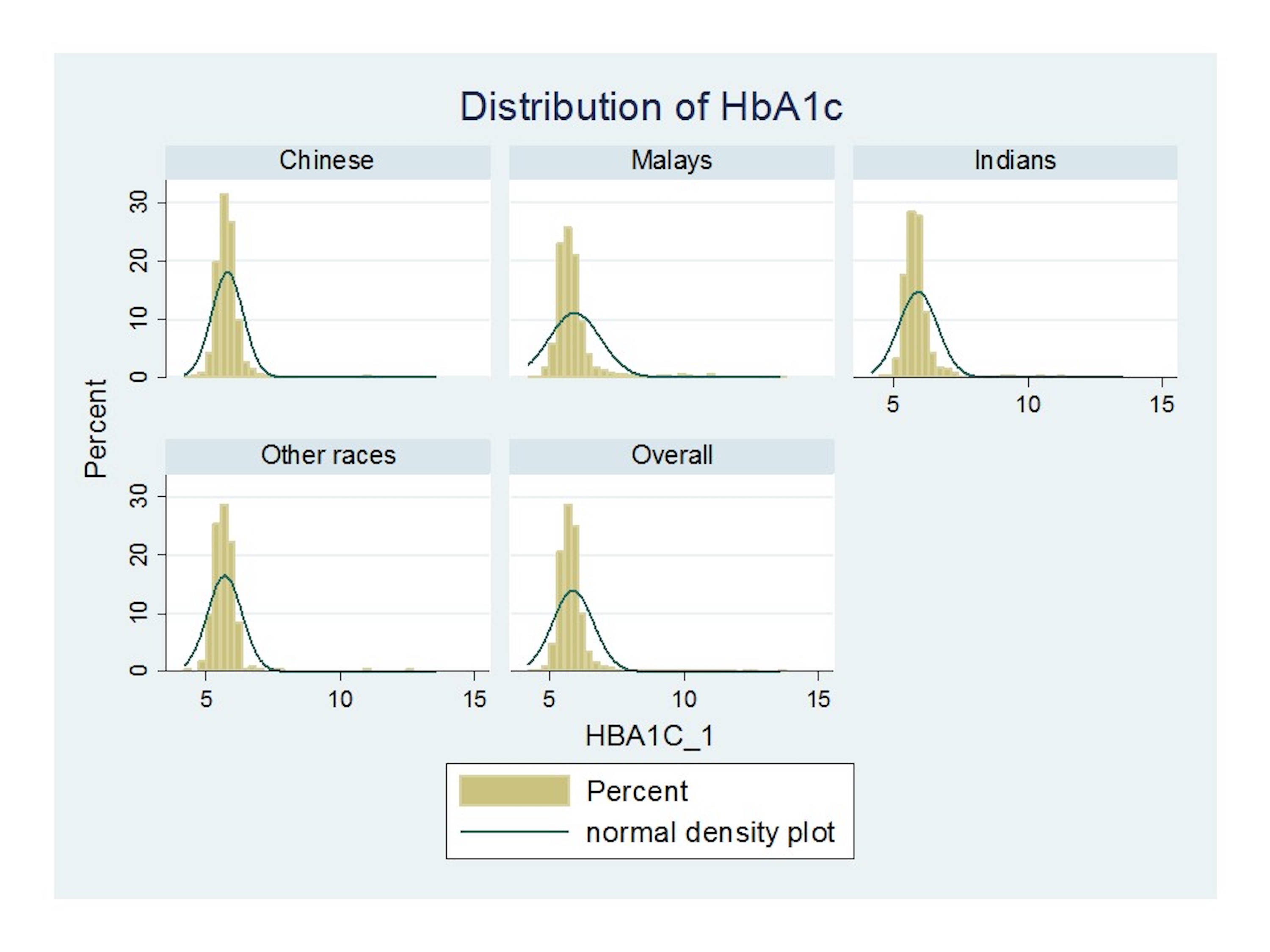

Supplement: Supplementary file 1 — Supplementary Information [file 41598_2018_29998_MOESM1_ESM.docx]
